# Mr. Golding's Case of Small-Pox after Vaccination

**Published:** 1807-06

**Authors:** W. Golding

**Affiliations:** Reading


### *To the Editors of the Medical and Physical Journal.*

GENTLEMEN,

A Case of small-pox, after vaccination, having lately occurred in the course of my practice, I think it my duty to lay before the public a faithful description of the same, though I would by no means wish to be conside ed as an anti-vaccinist, or endeavouring by this publication to depreciate the merits of Dr. Jenner's valuable discovery.—Soon after his first Treatise appeared, I introduced it into practice, and have repeatedly witnessed its good effects as a preventive against the small-pox.

I am, &c.

*Reading, March,* 1807. W. GOLDING.

---

IN June, 1802, by the desire of Mrs. B—, wife of a respectable farmer in this neighbourhood, I inoculated five of her daughters with the cow-pox. The disease, in all of them, went through the regular stages with the characteristic marks of the vaccine vesicle; and the indisposition which each experienced, convinced me they had all received the genuine disease, and were safe from the contagion of the small-pox.

The unfavourable reports which have lately prevailed, respecting vaccination, rendering them very uneasy, as they feared they were not safe from infection, they requested to be inoculated with the small-pox; and having a patient with that disease, in the natural way, I inoculated them all with small-pox matter on Dec. 1, 1806.

A few

A few days after, going to the same village, I took the opportunity to call, and examine the appearance of their arms; it was the fifth day from the insertion of the matter, and they appeared to have all taken; but the arms of Sarah and Eliza were in a more forward state than the rest.

Dec. 13. Mrs. B. called this morning, to say, that Eliza had been very unwell for two or three days, complaining of pain in her head and back, with thirst, and the usual symptoms of fever. She did not think she had taken cold, but was certainly sickening for the small-pox. An opening mixture was ordered to be given, and repeated till the bowels were sufficiently evacuated. On going to rest, her mother observed several eruptions in different parts of her body.

Dec. 14. Heard this morning that she was considerably better, and many more eruptions were observable.

Dec. 15. Being anxious to acquaint myself with the nature of the eruption, I went over this morning, and examined them minutely. I shall insert each patient separately, with name, age, and state of the arm, as I found them.

Sarah, aged eighteen years. The efflorescence had extended itself considerably round the punctured part, which contained matter, but she had not experienced any constitutional affection. One of the men servants in the house, not having had the small-pox, was inoculated with matter from her on this day.

Harriot, aged sixteen years; Jane, aged thirteen years; and Caroline, aged eleven years; in each, the punctured part was surrounded by a small areola, but not near so extensive as Sarah's; nor had either of them experienced any indisposition. Eliza, aged nine years, who had been ill for three or four preceding days, was now much better; her arm appeared the same as her sister Sarah's, but she had about a hundred small pustules in different parts of her body; many of them contained a fluid, with which I inoculated another of the men servants. About 1 o'clock, P. M. a messenger came over, to desire me to go to Mr. B's immediately, as Eliza was taken much worse. Mrs. B. informed me, she began to complain early in the evening, and went to bed about eight o'clock; she then became more restless, and at times was not sensible; complained of her head, and had several rigors; she threw her arms very much about, and a peculiar rigidity affected both the upper and lower extremities, like that of a person in the agonies of death, which led her parents and sisters to conceive ceive that she was really dying. When I got there I found her more composed, and she answered the questions put to her very properly. Her tongue was white, countenance flushed, pulse quick; she complained particularly of her throat; and on examination, the tonsils were enlarged, and inflamed. She had had an evacuation in the course of the day. The pustules were increased in size, and many of them were surrounded by a red basis. She was directed to take a febrifuge draught every four hours, and her bowels to be kept open with the aperient mixture.

Dec. 16. She had got some rest towards morning, and I found her considerably better. The most troublesome complaint was her throat, which was in the same state as in the night; the pustules, though small, appeared filled. I sent her an astringent gargle, and directed the draught to be continued.

Dec. 17. She was up this morning, and better in every respect; the contents of the pustules put on the appearance of matter.

Dec. 20. The pustules began to diminish, and the arm to exsiccate. The men who were inoculated being from home, I was informed that both their arms had taken; and Mrs. B. of whom I have since inquired, informs me, they both sickened at the customary period, were ill for two or three days, and had a considerable number of pustules.

From the above Cases we may draw the following inferences. It is I believe a just observation, that too early an inflammation of the arm will defeat the intended purpose, and will prove the destruction of its own specific virus; and that in such cases the patient will not be safe from variolous infection. The Vaccinist will say, might not this be the cause of the failure, in the case of Eliza? But it should be noticed, they were all inoculated with the same matter; the progress of their arms was regular and characteristic, and each experienced constitutional affection. I have this circumstance to mention respecting Eliza, that after the disease had gone through its regular stages, and the habit had been affected, the part where the matter was inserted degenerated into what Dr. Jenner calls erysipelatous inflammation; the wound became deeper and larger, and a secondary constitutional affection was produced: but by the use of the saturnine poultice, the inflammation abated, the arm soon got well, and no further disease was observable. But I never heard or read of the secondary indisposition destroying the previous constitutional tutional affection, and thereby rendering the patient liable to the influence of the small-pox.

I conceive the complaint she experienced during the eruptive process, to have originated from cynanche tonsillaris, and not from the effects of variola; for, exclusive of the former disease, she would have had the small-pox in a mild way.

That no doubt might remain, respecting the nature of the eruption, one of the men servants, who had not had the small-pox, was infected from one of the pustules, and had the disease regularly. We likewise find, that the matter taken from Sarah's arm, though she experienced no indisposition, communicated the small-pox to another of the men servants.

